# Fibrosarcoma of the corpus cavernosum: case report and literature review

**DOI:** 10.1186/s12893-020-01006-0

**Published:** 2021-01-06

**Authors:** Ziwei Liu, Wenda Zou

**Affiliations:** 1 Department of Urology, Zhuzhou Hospital Affiliated to Xiangya School of Medicine, Central South University, ZhuZhou, China; 2 Department of Reproductive Medicine, Zhuzhou Hospital Affiliated to Xiangya School of Medicine, Central South University, South Changjiang Road, Tianyuan District, ZhuZhou, 412007 China

**Keywords:** Fibrosarcoma, Corpus cavernosum, Chemotherapy, Local excision

## Abstract

**Background:**

Fibrosarcoma is a very rare tumor that arises from fibrous tissue. Less than 5% of fibrosarcoma originate from the urogenital tract. Penile fibrosarcoma, even more rare, is characterized by pain, enlargement, penile erection and urinary tract obstruction. To our knowledge, this is the second reported case named “fibrosarcoma of the corpus cavernosum”.

**Case presentation:**

A 51-year-old male presented with a 1-month history of penis pain during erection. CT scan showed a soft tissue mass arising from the proximal part of the penis. We diagnosed it as penile sarcoma, performing local excision. The postoperative pathological result was moderately differentiated fibrosarcoma. 3 months later, CT scan showed the recurrence of the tumor, and multiple metastases. Although he received chemotherapy, he died 10 months after surgery.

**Conclusions:**

Fibrosarcoma of the corpus cavernosum is rare and have poor prognosis. Total penile amputation may be the best treatment. The effects of chemotherapy are limited. No more effective treatment has been found for a disseminated disease to date.

## Background

Fibrosarcoma is a very rare tumor that arises from fibrous tissue. It generally involves the extremities and the trunk but rarely involves the genital area. There are only very few cases of penile fibrosarcoma in the published literature via a search of PubMed. The age distribution ranges from 2 to 72 years old. The best treatment for fibrosarcoma is complete excision of the tumor and total penile amputation is recommended. However, we report a case of fibrosarcoma of the corpus cavernosum which was treated by local excision, and summarized the current data regarding etiology, diagnosis, and management from literature.

## Case presentation

**Fig. 1:**
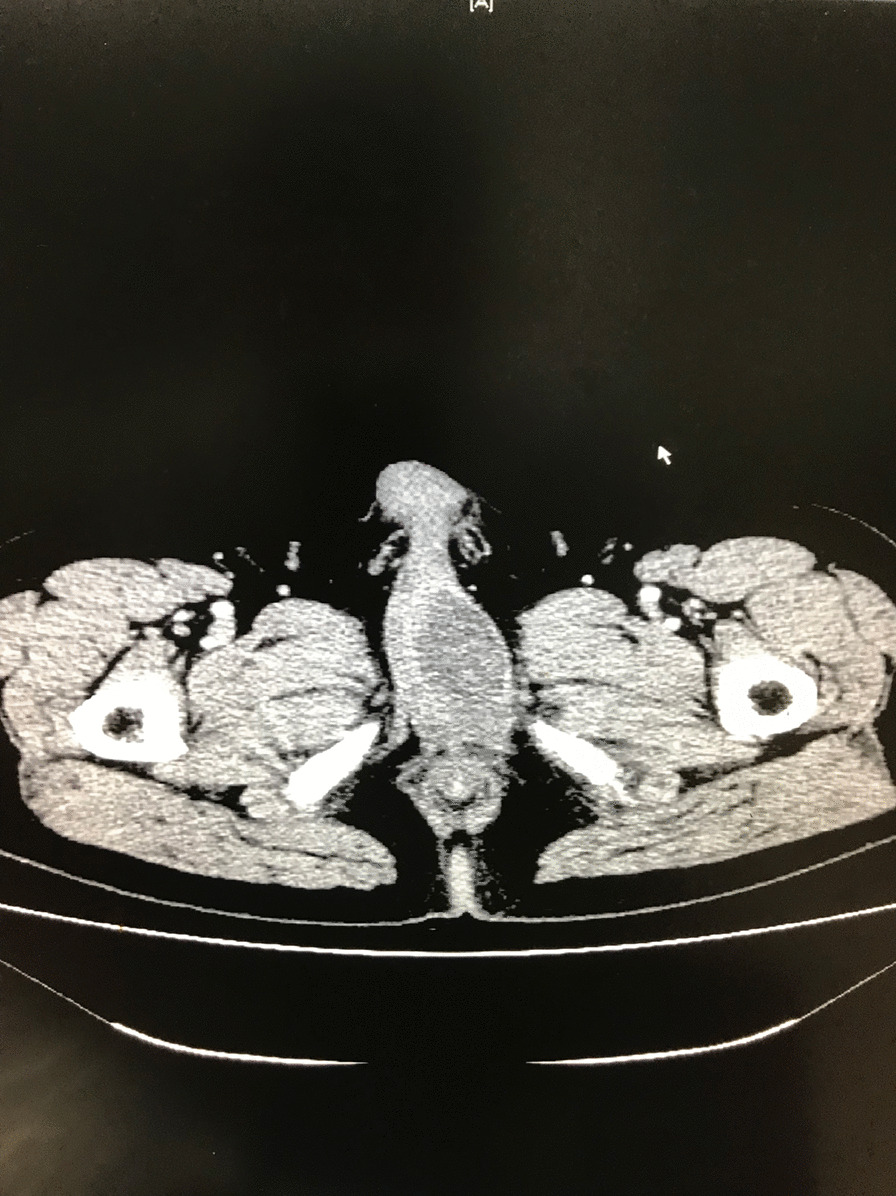
Computed tomography imaging. A well-defined, irregular, and heterogeneously enhancing soft tissue mass arising from the proximal part of the penis

**Fig. 2:**
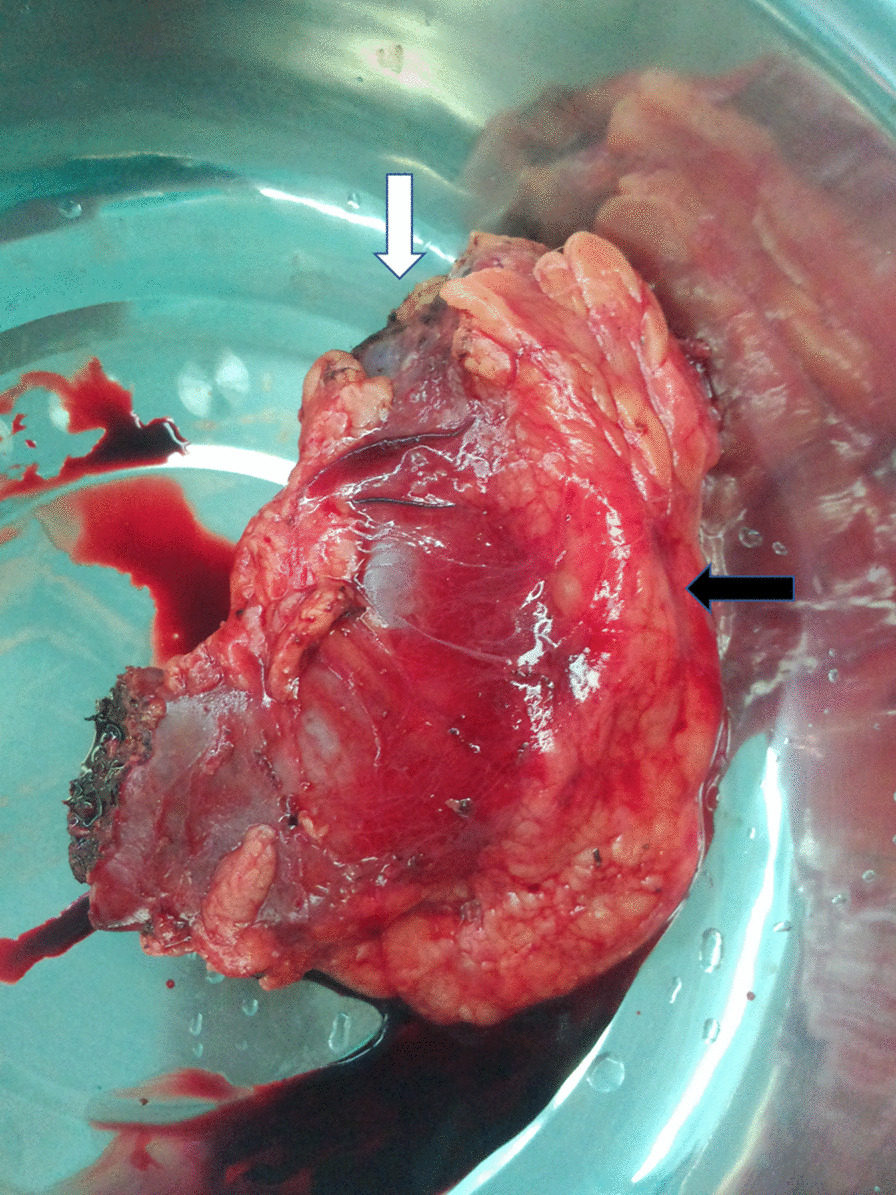
Proximal margin of the tumor (white arrow) and the surrounding fat (black arrow)

**Fig. 3:**
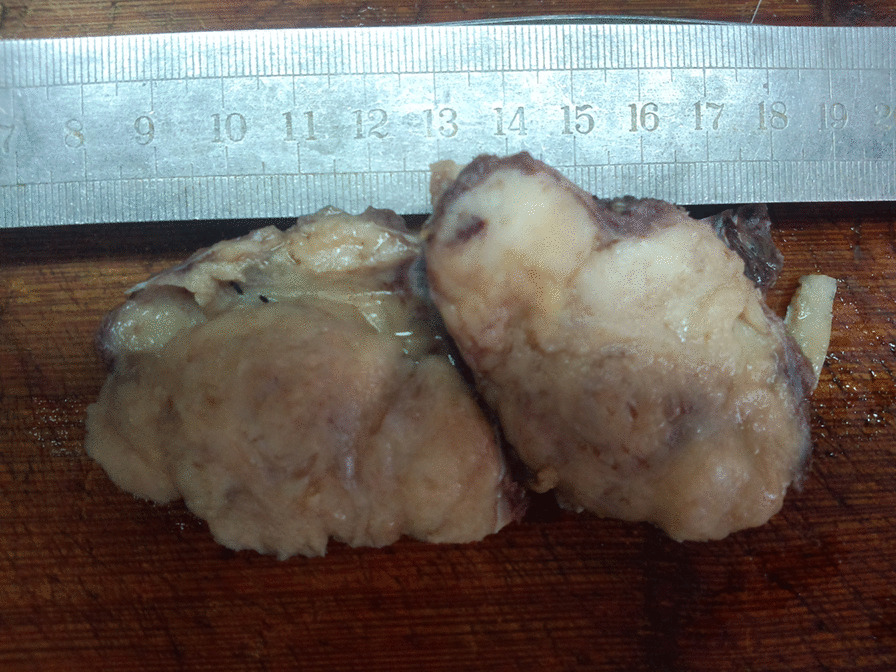
Section of the tumor

**Fig. 4:**
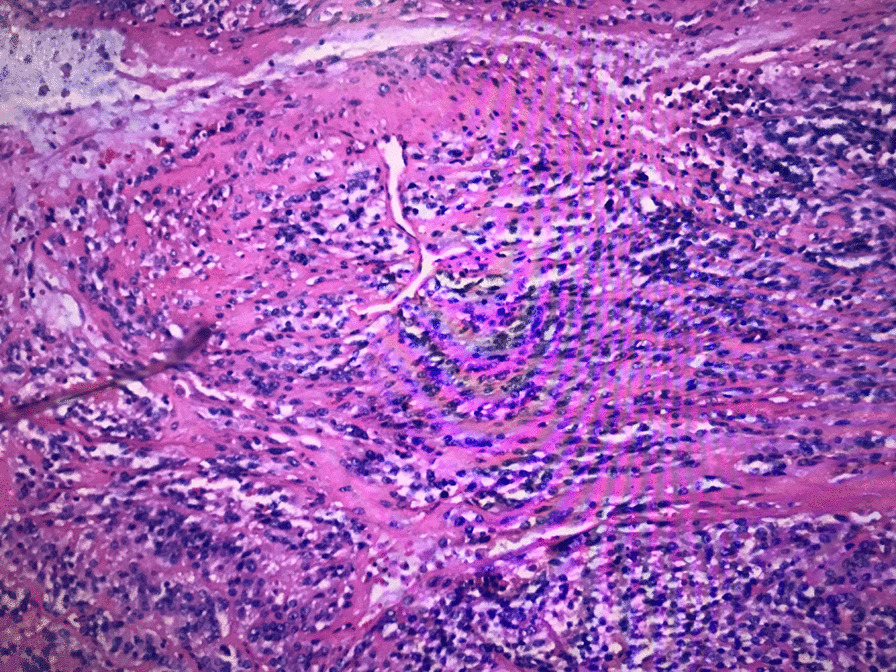
Fibrosarcoma of corpus cavernosum (HE, 100X)

**Fig. 5:**
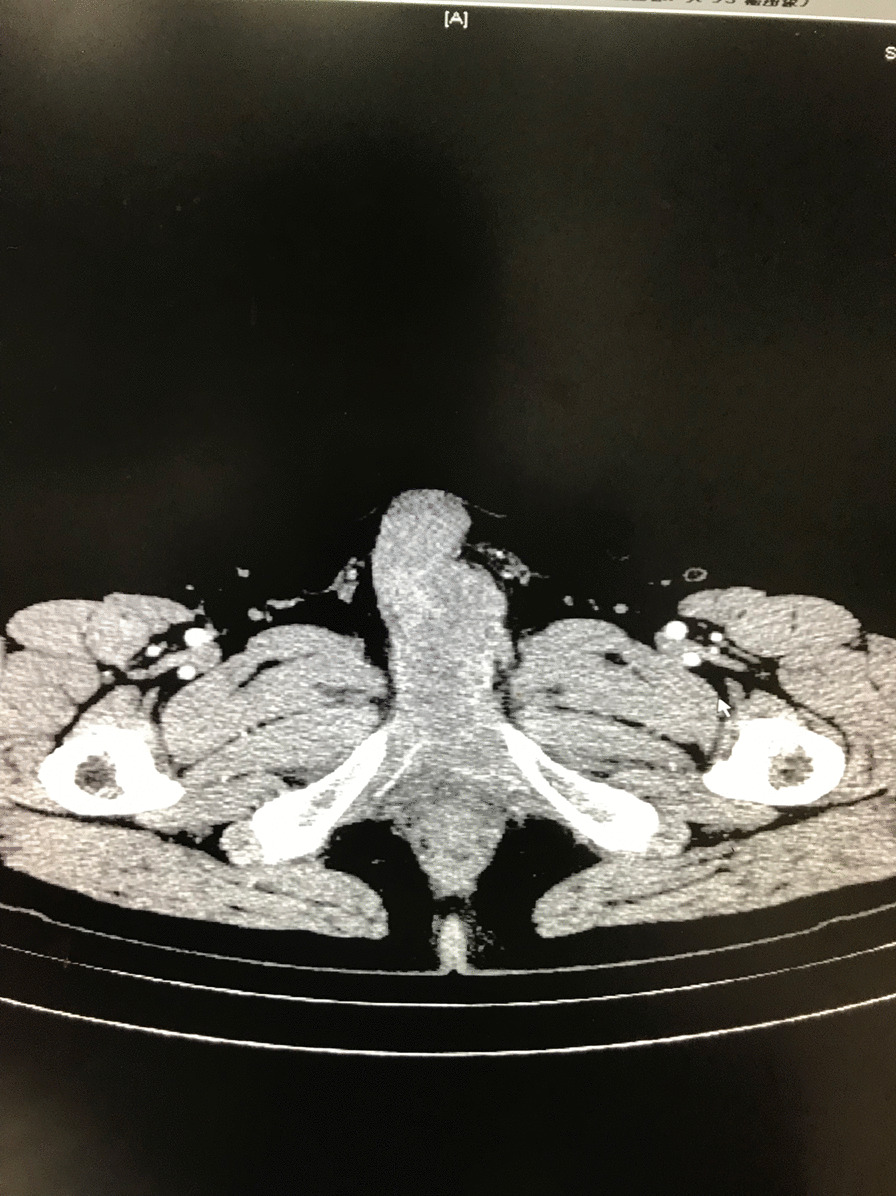
Computed tomography imaging. 3 months after surgery

**Fig. 6:**
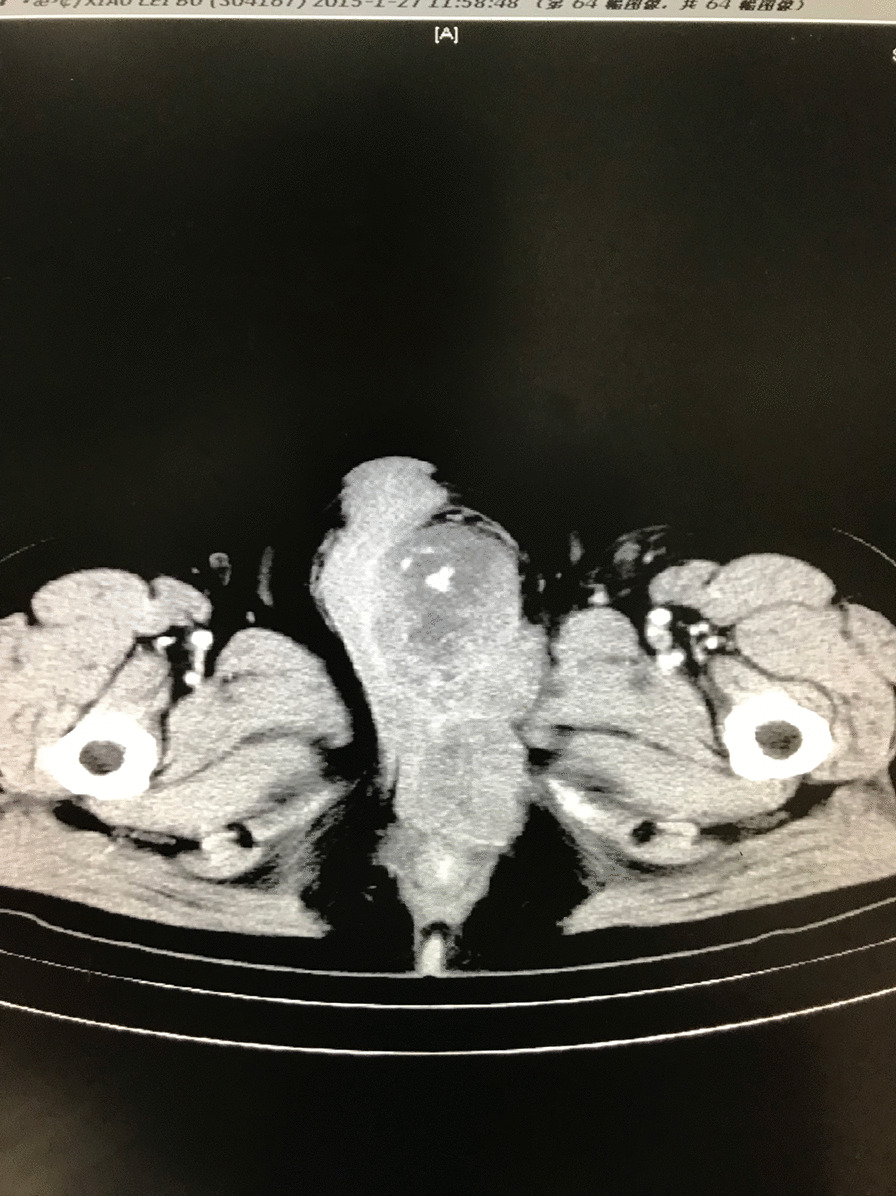
Computed tomography imaging. 5 months after surgery

A 51-year-old male presented with a 1-month history of penis pain during erection. He had a slight painful urination, no frequent urination, urgency, hematuria. His BMI is 21.1. There is no such disease in his family. He had not received any relevant treatment. Examination of his genital area revealed the left of the penile base had a fixed mass which was hard, with unclear boundary, tenderness, measuring approximately 7.0 cm in diameter. There were no palpable enlarged lymph nodes in the bilateral inguinal region. Hemoglobin level was 123 g/L, microscopic urine analysis was normal, as were radiological examinations of the chest and abdomen. Doppler ultrasonography showed deep perineal solid mass with irregular shape and visible blood flow signal inside. Computed tomography (CT) scan of abdomen and pelvis showed the presence of a well-defined, irregular, and heterogeneously enhancing soft tissue mass arising from the proximal part of the penis, and measuring 3.2 cm in width by 7.2 cm in length, no enlarged lymph node was seen in the groin and pelvis (Fig. 1). CT value of the mass was about 33 HU and 40–48 HU after enhancement. Urethral cystoscopy suggested that the mucosa of urethra and bladder was normal. At that time, we diagnosed that it might be a penile sarcoma (cT_2_N_0_M_0_) with poor prognosis, and recommended him penectomy if the intraoperative frozen section diagnosis was sarcoma or other malignant tumors. Influenced by traditional Chinese male culture, he and his family refused penectomy. Unfortunately, the frozen pathological section indicated sarcoma. So, we recommended penectomy again during the operation, but his family rejected it for the second time. Then we had to perform local excision. We saw a hard tumor on the left side of the corpus cavernosum in the surgery, and had to remove part of the Buck fascia because of the adhesion, then stitched the tunica albuginea (Figs. 2 and 3). The frozen section of the base was negative. The postoperative pathological section, which consistent with moderately differentiated fibrosarcoma, showed sheets of spindle-shaped, solidly packed cells arranged in bundles, with the involvement of surgical margins (Fig. 4). We recommended him to undergo adjuvant radiotherapy (DT50-60 Gy) or 3 cycles of a chemotherapy regimen consisting of docetaxel and gemcitabine after surgery, but he rejected and was discharged from the hospital on the 7th day of postoperative. About 3 months later, he was admitted to the hospital again due to recurrence. CT showed that a mass could be seen on the left side of the proximal part of the penis, measuring 3.5 cm in width by 9.7 cm in length, and CT value was the same as before, inguinal and pelvic lymph nodes metastasis (Fig. 5). Considering the recurrence of the tumor and the difficulty of resection, the patient was transferred to the department of oncology for chemotherapy. 3 weekly cycles of a chemotherapy regimen consisting of docetaxel and gemcitabine were begun, to shrink the tumor. After the chemotherapy, CT showed that the size of the mass was about 13.5 cm long and 4.7 cm wide, and it had invaded the internal muscle of bilateral obturator foramen and pelvic floor fascia, damaged the pubic bone and ischial bone, and multiple metastases occurred in the lung and liver (Fig. 6). When we were ready to adjust the chemotherapy plan, he refused further treatment and died 10 months after surgery.

## Discussion and conclusions

Penile tumor is rare, and the incidence in developed countries ranges from 0.6 to 1/100,000 patients [1]. Epidermoid carcinoma is the predominant histologic type. Sarcoma of the penis is rare. It is estimated that less than 5% of soft tissue sarcomas derived from the urogenital tract, and they only account for 1–2% of tumors in the system [2].

According to the tissues from which they originate, the penile sarcoma can be divided into superficial and deep-seated [3]. Superficial lesions occur in the integumentary supporting structures, mostly located in the distal parts of the penis, showing a low-grade malignancy, rarely invade deep tissues and distant metastases. In contrast, deep-seated lesions arise from the corporeal body supporting structures, including lesions originating from the glans, lesions involving the cavernous body and/or cavernous smooth muscle, usually present more invasiveness and poorer prognosis [4, 5].

Fibrosarcoma of corpus cavernosum is derived from the supporting structure of the corpus cavernosum and classified as the deep-seated sarcoma. Clinical manifestations mainly include subcutaneous mass, penis pain and enlargement, penile erection and urinary tract obstruction. A review of the literature showed that 3 cases of epithelioid sarcoma occurred which were found to be causing pain during erection as in our case [6–8]. Moreover, Moore et al. [9] had reported a penile sarcoma masquerade as a Peyronie's disease.

Some features related to penile sarcoma recurrence and metastatic dissemination patterns have been well confirmed. Local recurrence is characteristic of sarcomas [10], and even superficial lesions should be considered for total penectomy [11]. On the other hand, local metastases are rare. Lymphadenectomy is not recommended unless adenopathy is palpable [12, 13]. Distant metastases are also uncommon, with the most common distant metastases being to the lungs, liver, and brain [14].

The treatment of fibrosarcoma of corpus cavernosum includes surgical resection, chemotherapy, and radiotherapy. Total penile amputation has been advised for tumors of deep corporeal origin. In 1932, Evans [15] reported the first surgical treatment of fibrosarcoma of corpus cavernosum, resecting the tumor and part of the cavernosa. Russo et al. [16] reported 43 patients with urogenital sarcoma which complete tumor resection was done in 72% of the cases, and 58% had disease-free margins. Survival rates after 3 and 5 years were 55% and 40% respectively. Fetsch et al. [17] reported 14 cases of leiomyosarcoma and concluded that small, less than 2-cm lesions were best managed with local resection whereas deeper-seated tumor often requires partial or total amputation. Deep lesions at the base of the penis have the worst prognosis. In our case, we recommended a penectomy. Because of the positive margin after local excision, local recurrence and regional metastasis soon followed. Antunes [1] report 3 cases of deep-seated primary sarcomas of the penis, although surgical margins were free from the tumor in 2 of the 3 cases, the patients quickly evolved to disseminated disease.

Radiotherapy has been used to control the local diseases in patients with unresectable tumors and positive margins, but the role of radiotherapy is limited. There have been contrasting reports of success with radiotherapy versus secondary fibrosarcoma due to radiation-induced neoplasia [18]. In this case the patient did not receive radiotherapy, this is a limitation. While chemotherapy was used for disseminated disease. Taib et al. [19] report a case in which partial recovery of the penile structure was achieved after chemotherapy. Russo et al. found that no patient with the disseminated disease was fully responsive to the treatment of several chemotherapy regimens [16]. The patient in this case did not respond to the chemotherapy regimen, and the tumor continued to progress.

We can predict the biological behavior of penile sarcomas by some previously described prognostic factors [16]. These factors include the size of the lesion (larger or smaller than 5 cm), the expansion of the invasion (superficial or deep-seated), complete lesion resection, whether there is metastatic disease, and the expression of retinoblastoma genes [20].

It is concluded that from this case study fibrosarcoma of the corpus cavernosum is rare, usually progressed rapidly, and has a poor prognosis due to the deep-seated lesion. Local excision is inadequate. Total penile amputation with negative margins may be the best treatment. Chemotherapy can be used as adjuvant therapy, but their effects are limited. No more effective treatment has been found for a disseminated disease to date.

## Data Availability

The datasets used and/or analysed in the current study are available from the corresponding author upon reasonable request.

## Abbreviations

CT: Computed tomography
HU: Hounsfield unit
HE: Hematoxylin–eosin staining

## References

[1] Antunes AA, Nesrallah LJ, Goncalves PD, Ferreira YA, Campagnari JC, Srougi M (2005). Deep-seated sarcomas of the penis. Int Braz J Urol.

[2] de Kernion JB, Tynberg P, Persky L, Fegen JP (1973). Proceedings: carcinoma of the penis. Cancer.

[3] Pratt RM, Ross RT (1969). Leiomyosarcoma of the penis. A report of a case. Br J Surg.

[4] Coindre JM, Trojani M, Contesso G, David M, Rouesse J, Bui NB, Bodaert A, De Mascarel I, De Mascarel A, Goussot JF (1986). Reproducibility of a histopathologic grading system for adult soft tissue sarcoma. Cancer.

[5] Katona TM, Lopez-Beltran A, MacLennan GT, Cheng L, Montironi R, Cheng L (2006). Soft tissue tumors of the penis: a review. Anal Quant Cytol Histol.

[6] Pueblitz S, Mora-Tiscareno A, Meneses-Garcia AA, Lozano-Lozano G, Ocampo-delCarpio O (1986). Epithelioid sarcoma of penis. Urology.

[7] Iossifides I, Ayala AG, Johnson DE (1979). Epithelioid sarcoma of penis. Urology.

[8] Sirikci A, Bayram M, Demirci M, Bakir K, Sarica K (1999). Penile epithelioid sarcoma: MR imaging findings. Eur Radiol.

[9] Moore SW, Wheeler JE, Hefter LG (1975). Epitheloid sarcoma masquerading as Peyronie's disease. Cancer.

[10] Dehner LP, Smith BH (1970). Soft tissue tumors of the penis. A clinicopathologic study of 46 cases. Cancer.

[11] Webber RJ, Alsaffar N, Bissett D, Langlois NE (1998). Angiosarcoma of the penis. Urology.

[12] Pow-Sang MR, Benavente V, Pow-Sang JE, Morante C, Meza L, Baker M, Pow-Sang JM (2002). Cancer of the penis. Cancer Control.

[13] Gamoudi A, Bougrine F, Dhiab T, Mamhlouf R, Chebi Goucha A, Khomsi F, Hechiche M, Benna F, Boussen H, el May A (2002). Leiomyosarcoma of the penis: a case report and review of the literature. Tunis Med.

[14] Antoneli CB, Novaes PE, Alves AC, Cardoso H, Lopes A (1998). Rhabdomyosarcoma of the penis in a 15-month-old boy. J Urol.

[15] Evans A (1932). Fibrosarcoma of the corpus cavernosum. Proc R Soc Med.

[16] Russo P, Brady MS, Conlon K, Hajdu SI, Fair WR, Herr HW, Brennan MF. Adult urological sarcoma. J Urol. 1992;147(4):1032–36; discussion 1036–37. 10.1016/s0022-5347(17)37456-6 1552580

[17] Fetsch JF, Davis CJ, Miettinen M, Sesterhenn IA (2004). Leiomyosarcoma of the penis: a clinicopathologic study of 14 cases with review of the literature and discussion of the differential diagnosis. Am J Surg Pathol.

[18] Hamm CM, Pyesmany A, Resch L (1997). Case report: congenital retroperitoneal fibrosarcoma. Med Pediatric Oncol.

[19] Taib F, Mohamad N, Mohamed Daud MA, Hassan A, Singh MS, Nasir A (2012). Infantile fibrosarcoma of the penis in a 2-year-old boy. Urology.

[20] Cance WG, Brennan MF, Dudas ME, Huang CM, Cordon-Cardo C (1990). Altered expression of the retinoblastoma gene product in human sarcomas. N Engl J Med.

